# A Rare Case of Gastric Metastasis in Ewing's Sarcoma of the Femur

**DOI:** 10.1155/2019/2870302

**Published:** 2019-05-14

**Authors:** Krishna Amulya Koti, Selvamani Backianathan, Patricia Sebastian, Leni G. Matthew, Mithun Raam, Dipti Masih

**Affiliations:** ^1^Department of Radiation Oncology, Christian Medical College, Vellore, India; ^2^Department of Child Health, Christian Medical College, Vellore, India; ^3^Department of General Surgery, Christian Medical College, Vellore, India; ^4^Department of Pathology, Christian Medical College, Vellore, India

## Abstract

The stomach is a very unusual site of metastasis. Published reports on metastatic lesion in the stomach is generally limited to single case reports and case series. Gastric metastasis in an Ewing's sarcoma is extremely rare and has been reported in English literature but once to our knowledge. We present a case report of Ewing's sarcoma of the right proximal femur metastasizing to the stomach. A young female treated for Ewing's sarcoma of the femur in 2012 presented with gastric metastasis after four years of disease-free interval. She was treated with irinotecan-based chemotherapy followed by total gastrectomy with esophagojejunal anastomosis and radiation therapy. At one-year follow-up, she was disease free.

## 1. Background

Ewing's sarcoma family of tumours (EFTs) comprises of a spectrum of tumours with a common histological, immunohistochemical, and chromosomal translocations. It includes Ewing's sarcoma, extra-skeletal Ewing's sarcoma, Askin's tumour, and peripheral primitive neuroectodermal tumour [1]. One of the major prognostic factors for survival is the presence of metastasis. The lung and other bones are common sites of metastasis; other sites being unusual and rare. The stomach is an uncommon site for primary Ewing's sarcoma; it is a very rare site for metastasis [2]. We report a case of stomach metastasis in a patient treated for Ewing's sarcoma of the extremity. To our knowledge, only one case has been reported in literature of Ewing's sarcoma with stomach metastasis so far.

## 2. Case Presentation

A 14-year-old girl presented to our hospital in February 2012 with complaints of painful swelling of the right thigh for 8 months and difficulty in walking. There was no history of trauma, fever, or other constitutional symptoms. Magnetic resonance imaging (MRI) showed right proximal femur lesion. Biopsy from the same was reported as malignant small round cell tumour suggestive of Ewing's sarcoma. Tumour cells were immunopositive for CD99 and negative for CD3, CD79a, MPO, and desmin. There was no systemic metastasis on further evaluation. She received 6 cycles of chemotherapy with vincristine, ifosfamide, doxorubicin, and etoposide (VIDE) followed by a wide local excision of the tumour with endoprosthesis insertion on 06/09/2012. Surgical specimen histopathology examination did not show viable tumour. Margins were free of tumour. She had received further 8 cycles of vincristine, ifosfamide, and actinomycin D (VAI). She was on regular follow-up. In 2016, she had presented with abdominal pain with low-grade fever and unquantified weight loss. Ultrasound abdomen showed epigastric mass measuring 8 cm × 7.5 cm.

### 2.1. Investigation

**Figure 1:**
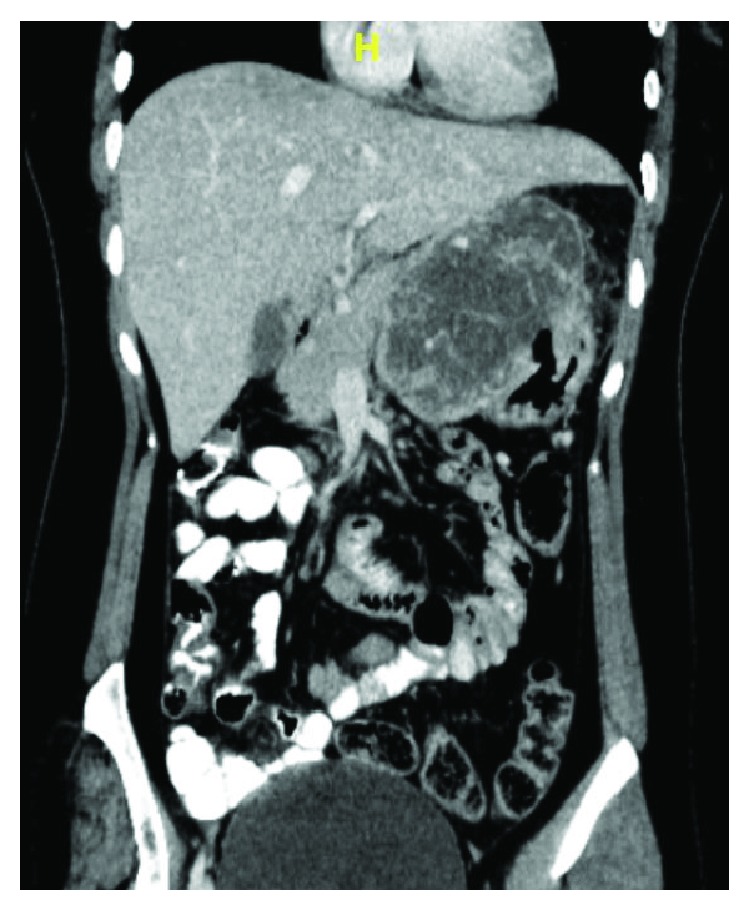
CT coronal section of the abdomen and pelvis showing a well-defined solid-cystic mass along lesser curvature of the stomach.

On computed tomography (CT) of the abdomen and pelvis (Figure 1), a 9 cm × 11 cm × 10 cm (anteroposterior×craniocaudal×transverse) well-defined exophytic mixed solid-cystic mass was located along the lesser curvature of the stomach bulging into the superior and inferior recesses of the lesser sac. The lesion had thick, irregular walls and enhancing solid components interspersed within. Multiple enhancing intratumoral vessels were present. Mass was seen abutting the inferior surface of the left lobe of the liver superiorly, the pancreatic body posteroinferiorly with no definite invasion. There was no loss of fat plane with adjacent structures. No lymphadenopathy or ascites was noted.

**Figure 2:**
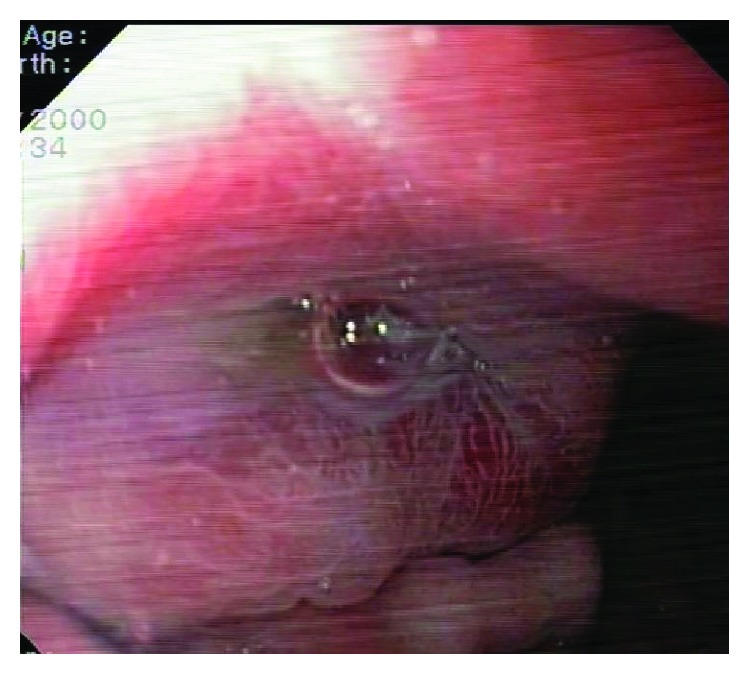
Gastroscopy showing mucosal bulge along the lesser curvature.

Upper gastrointestinal endoscopy showed a mucosal bulge along the lesser curvature of the body of the stomach and proximal antrum (Figure 2). A single deep mucosal ulcer was noted in the mid-body along the lesser curvature with surrounding mucosal edema and erythema and friability. Clinical differentials considered were gastrointestinal stromal tumour (GIST), lymphoma, and adenocarcinoma. Metastasis was considered unlikely in view of the stomach being a rare site. However, biopsy from the lesion was reported as malignant round cell tumour, immunopositive for CD99 and FLI-1. Immunohistochemistry marker for CK, DOG, desmin, and TdT was negative ruling out epithelial tumours, GIST, rhabdomyosarcoma, and lymphoblastic lymphomas, respectively. Bone marrow examination did not reveal metastatic infiltration. No suspicious lesions were seen on bone scintigraphy. CT thorax was negative for lung metastasis.

### 2.2. Treatment

After discussion with the multidisciplinary tumour board (MDT), it was considered to be a metastatic lesion from previous Ewing sarcoma and it was planned to offer her systemic chemotherapy followed by assessment for gastrectomy. She received 6 cycles of chemotherapy with irinotecan and temozolomide till 07/03/17.

**Figure 3:**
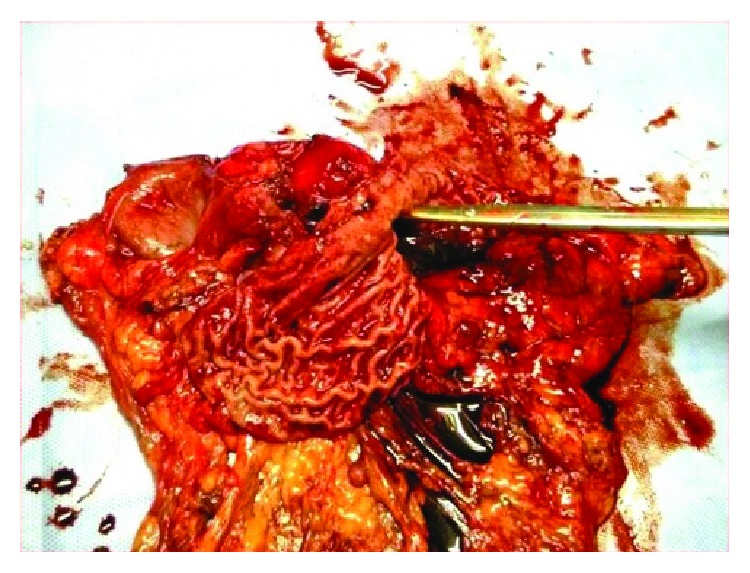
Surgical specimen shows the stomach with tumour.

She underwent total gastrectomy with end-to-side esophagojejunal stapled anastomosis and feeding jejunostomy on 07/04/17 (surgical specimen is seen in Figure 3). At surgery, an exophytic lesion along the lesser curvature of the stomach with mucosal involvement adherent to the transverse mesocolon was found. There was no ascites or liver or nodal metastasis.

**Figure 4:**
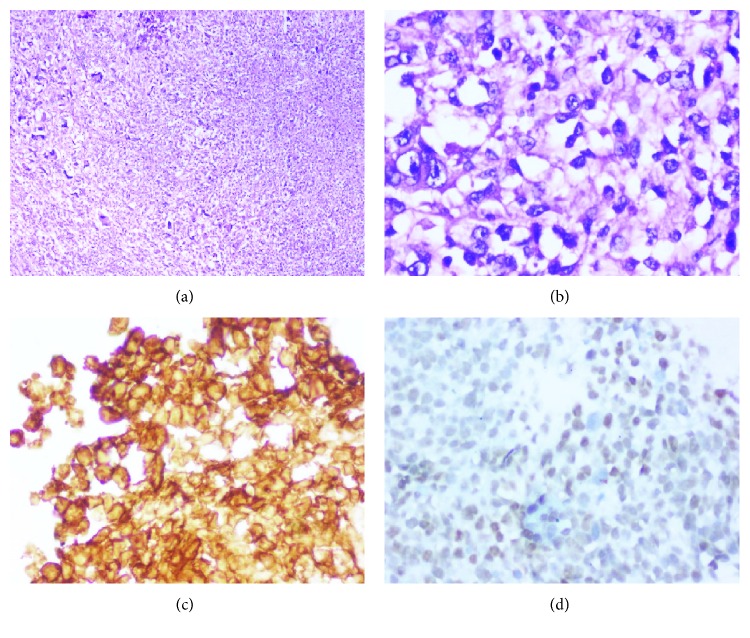
Histopathological images. (a) Hematoxylin and eosin (H&E) 200x magnification. Photomicrograph shows diffuse sheets of tumour cells. (b) H&E 400x uniform cells with round nuclei; small nucleoli with scant-to-moderate clear cytoplasm. (c) Immunohistochemistry (IHC) staining for CD99 shows crisp cytoplasmic membrane staining. (d) IHC for FLI-1 shows moderate nuclear staining in the tumour cells.

Surgical specimen histopathological examination was reported as malignant round cell tumour (viable tumour: 80-85%), consistent with Ewing's sarcoma. Hematoxylin and eosin staining showed characteristic uniform cells with the round nuclei, small nucleoli with scant-to-moderate clear cytoplasm (Figures 4(a) and 4(b)). Margins were free of tumour. Omentum was noted to have focal tumour deposit. Eight perigastric lymph nodes with reactive hyperplasia. On immunohistochemistry, the tumour cells were diffusely positive for CD99 (Figure 4(c)). In view of poor response to chemotherapy, following discussion with the multidisciplinary tumour board, it was decided to rule out anaplastic large-cell lymphoma. Pathologist had a re-look at her biopsy and surgical specimen slides. Lymphoma and GIST IHC panel was negative. FLI-1 was positive on immunohistochemistry (Figure 4(d)). Reverse transcription polymerase chain reaction (RT-PCR) on the gastric resection specimen was negative for EWS-FLI-1 types 1 and 2, EWS-ERG, and EWS-FEV translocations. After ruling out epithelial tumours, rhabdomyosarcoma, lymphoma, and GIST, the likelihood of having a RT-PCR negative Ewing's sarcoma that is known to have an incidence of 10-15% was considered.

It was decided to offer her radiation therapy in view of poor response to chemotherapy and omental deposit by the MDT team. A renogram was performed prior to initiation of RT, and an informed consent was obtained. She received intensity-modulated radiotherapy to a dose of 50.4 Gy in 28 fractions, delivered once a day over 5 and a half weeks. She received further chemotherapy with irinotecan and temozolomide till July 2017.

At the last follow-up in March 2018, the patient was doing well without evidence of the disease.

## 3. Discussion

Ewing's sarcoma was first described as diffuse endothelioma of the bone by James Ewing in 1921. It is the second most common primary bone malignancy in children and adolescents [3]. Long bones are the usual sites of presentation in Ewing's sarcoma with the femur being the most common. Ewing's sarcoma and peripheral primitive neuroectodermal tumours were considered different entities in the past. However, recent cytogenetic and molecular studies suggest that they indeed are two ends of the same spectrum of tumours known as “Ewing sarcoma family of tumours (ESFT)” [4]. The most common chromosomal translocation is t(22;11)(q24;q12) that results in *EWS-FLI-1* fusion, seen in 85-90% of the ESFT. The remainder of the cases (approximately 15%) involves fusion of the *EWS* or *FUS* gene with a gene closely related to *FLI* like *ERG*, *FEV*, *ETV1*, and *ETV4* [5]. As RT-PCR using commercial primers is limited to only the most prevalent *EWSR1* fusion transcript, the rarer translocations associated with ESFT that could go undetected could be the likely explanation for our case being negative for the pathognomonic translocation.

Clinically, it is an aggressive tumour and with current multimodality approach (chemotherapy, surgery, and/or radiation), the 5-year survival rates for nonmetastatic disease ranges from 50 to 70% [6]. Incidence of metastasis is reported to be 20-30%, and the 5-year survival rate has improved from 20% to 30-40% with effective multidrug chemotherapy regimens in metastatic disease [7]. The lung and bones are the most common sites of metastasis. Patients with isolated metastasis to the lung were found to have better prognosis over patients with nonpulmonary metastatic sites [8].

We report a case of Ewing's sarcoma with stomach metastasis. Gastric metastasis is very rare and was reported in very few breast malignancies and malignant melanomas [9, 10]. Few case reports of primary extraosseous Ewing's sarcoma of the stomach were described [11]. However, there is extreme paucity of literature on the stomach being a metastatic site for Ewing's sarcoma. To our knowledge, only one other case of stomach metastasis from Ewing's sarcoma was reported in English literature [12].

## 4. Conclusion

When a tumour is encountered during follow-up of Ewing's sarcoma, metastasis should be considered as one of the diagnostic differentials.
